# Identification of the ER-resident E3 ubiquitin ligase RNF145 as a novel LXR-regulated gene

**DOI:** 10.1371/journal.pone.0172721

**Published:** 2017-02-23

**Authors:** Emma C. L. Cook, Jessica K. Nelson, Vincenzo Sorrentino, Duco Koenis, Martina Moeton, Saskia Scheij, Roelof Ottenhoff, Boris Bleijlevens, Anke Loregger, Noam Zelcer

**Affiliations:** 1 Department of Medical Biochemistry, Academic Medical Center, University of Amsterdam, Meibergdreef 9, Amsterdam, The Netherlands; 2 Laboratory for integrative and systems physiology, EPFL, Lausanne, Switzerland; IRCCS Istituto Oncologico Giovanni Paolo II, ITALY

## Abstract

Cellular cholesterol metabolism is subject to tight regulation to maintain adequate levels of this central lipid molecule. Herein, the sterol-responsive Liver X Receptors (LXRs) play an important role owing to their ability to reduce cellular cholesterol load. In this context, identifying the full set of LXR-regulated genes will contribute to our understanding of their role in cholesterol metabolism. Using global transcriptional analysis we report here the identification of *RNF145* as an LXR-regulated target gene. We demonstrate that RNF145 is regulated by LXRs in both human and mouse primary cells and cell lines, and *in vivo* in mice. Regulation of *RNF145* by LXR depends on a functional LXR-element in its proximal promotor. Consistent with LXR-dependent regulation of *Rnf145* we show that regulation is lost in macrophages and fibroblasts from *Lxrαβ*^*(-/-)*^ mice, and also *in vivo* in livers of *Lxrα*^*(-/-)*^ mice treated with the LXR synthetic ligand T0901317. RNF145 is closely related to RNF139/TRC8, an E3 ligase implicated in control of SREBP processing. However, silencing of *RNF145* in HepG2 or HeLa cells does not impair SREBP1/2 processing and sterol-responsive gene expression in these cells. Similar to TRC8, we demonstrate that RNF145 is localized to the ER and that it possesses intrinsic E3 ubiquitin ligase activity. In summary, we report the identification of RNF145 as an ER-resident E3 ubiquitin ligase that is transcriptionally controlled by LXR.

## Introduction

To maintain their quantities at adequate physiological levels, the synthesis and elimination of cholesterol and fatty acids are subject to coordinated transcriptional and post-transcriptional regulation [1]. Accordingly, disturbed regulation of their metabolism is detrimental to cellular function, and is systemically associated with development of metabolic and cardiovascular disease [2]. Orchestrating this delicate balance are two transcription factor families with opposing actions: the sterol regulatory element-binding proteins (SREBPs) and the liver-X receptors (LXRs) [3,4].

The three SREBP species (SREBP1a, SREBP1c, and SREBP2) are produced as ER membrane precursors, which undergo processing into their mature, transcriptionally active form when the concentration of cholesterol in the ER membrane drops to lower than 5% (w/w) [1,3,5]. As transcription factors, SREBPs induce the full set of genes required for cholesterol biosynthesis via the mevalonate pathway, as well as expression of the low-density lipoprotein receptor *(LDLR)* to promote cellular uptake of low-density lipoprotein-derived cholesterol [3,6]. Additionally, SREBP1a/c increase the levels of the rate-limiting enzymes in fatty acid synthesis, and induction of *SREBP1c* by insulin signaling in the liver is recognized as a key step in hepatic lipid synthesis in the fed state [6,7]. In contrast to SREBPs, which are activated when cellular sterol levels decline, LXRs are sterol-responsive transcription factors that become activated when cellular sterol levels increase [4]. Activation of LXR is dependent on their engagement with their cognate ligands: intermediates of the cholesterol biosynthesis pathway and oxysterols [8–10]. Ligand-activated LXRs induce a genetic program aimed at reducing the cellular sterol load by promoting cholesterol efflux [11–13], limiting cholesterol uptake [14,15], and attenuating cholesterol biosynthesis [16]. As such, identifying the full set of LXR-regulated genes will contribute to our understanding of cellular lipid handling.

Reflecting the tight regulation of cellular lipid levels, multiple feedback mechanisms are in place to balance the LXR and SREBP pathways. These involve both transcriptional and post-transcriptional mechanisms reflecting nuclear and non-nuclear events. Amongst these, ubiquitylation–the covalent conjugation of ubiquitin to protein targets [17]—is rapidly emerging as an important facet of cellular lipid homeostasis [18]. Amongst others, ubiquitylation is implicated in controlling nuclear levels of LXR [19,20] and SREBPs [21] and induction of the Inducible Degrader of the LDLR (IDOL) prompts rapid lysosomal degradation of the LDLR [14]. An important cellular site where ubiquitylation and lipid metabolism intersect is the ER, which is predicted to contain at least 24 E3 ubiquitin ligases [22]. Although disputed, two of these E3 ligases, GP78 and RNF139/TRC8, have been described to mediate the rapid sterol-stimulated ER-associated degradation (ERAD) of 3-hydroxy-3-methyl-glutaryl-coenzyme A reductase (HMGCR), a rate-limiting enzyme in cholesterol biosynthesis [23–27]. Similarly, we have recently implicated the E3 ligase MARCH6 in regulating ERAD of HMGCR and Squalene Epoxidase (SQLE) [28,29]. SQLE catalyzes the second rate-limiting step in this process and commits the mevalonate pathway to cholesterol synthesis. However, the role of many of the other remaining E3 ligases in the ER, and their potential involvement in modulating lipid metabolism is unknown. Herein, using transcriptomics, we identify RNF145 as a direct transcriptional target of LXRs. We provide evidence that RNF145 is localized to the ER, possesses E3 ubiquitin ligase activity, and discuss its potential role in cellular lipid metabolism.

## Materials and methods

### Reagents

GW3965, LG100268, Actinomycin D, T0901317, and Bafilomycin A1 were obtained from Sigma. Simvastatin salt and MG-132 were purchased from Calbiochem. 22(*R)*-hydroxycholesterol and desmosterol were acquired from Steraloids. Lipoprotein deficient serum (LPDS) was prepared as previously reported and confirmed to contain no lipoproteins [30]. All other reagents were purchased from Sigma.

### Plasmids and expression constructs

The mouse Rnf145 cDNA was amplified from Hepa1-6 cells and cloned into expression plasmids by Gateway recombination (Invitrogen). The C537A mouse Rnf145 mutant was generated by site directed mutagenesis. A 1 Kb human RNF145 proximal promotor region (chr5:158.634.810–158.635.824) predicted to contain an LXR responsive element (LXRE) was amplified from HEK293 genomic DNA and cloned into pGL3 basic (Promega) as an NheI and HindIII fragment. Mutations in the predicted LXRE were introduced by site-directed mutagenesis with the wild-type plasmid as template. The minimal identified wildtype and mutant mRnf145 LXRE was cloned in tandem into pGL2 SV40 min promoter (a kind gift from Dr. Phil Barnett, AMC) with KpnI and NheI. pGL3-hABCA1-LUC was kindly provided by Prof. Herbert Stangl (University of Vienna, Austria). pTK-RLUC encoding Renilla luciferase was used as a transfection control. pcDNA Calnexin-mCherry was a kind gift from Dr. Volodymyr Korkhov (Paul Scherrer Institute, Switzerland). The pDest527 N’-His_6x_ tag destination vector was a kind gift of Dr. D. Esposito (Frederick National Laboratory for Cancer Research, USA). For generation of recombinant RNF145 RING protein the RING domain region of wild type human RNF145 was amplified by PCR (from codon 1650 of the RNF145 cDNA which corresponds to amino acid 550) and cloned into pDest527 using Gateway cloning (Invitrogen). All plasmids used were isolated by CsCl_2_ gradient centrifugation. DNA sequencing was used to verify the correctness of all the constructs used in this study.

### Purification of recombinant RNF145 RING domain

His_6x_-tagged hRNF145 RING protein was produced in the bacterial RIPL strain (Novagen). Bacteria were grown in Luria Broth (LB) (Sigma) at 37°C to an *OD*_600_ of 0.6 and induced with 1 mm isopropyl 1-thio-β-d-galactopyranoside for 4 h. Bacterial pellets were collected and lysed in lysis buffer (50 mM Tris-HCl, pH 7.6, 0.5 M NaCl, 5 mM imidazole, and 1 mM DTT) supplemented with protease inhibitors and sonicated on ice to disrupt the cells. Debris was removed by centrifugation, and the cleared lysates were loaded onto HisTrap HP columns (GE Healthcare) coupled to an ÄKTAprime Plus protein purification system (GE Healthcare). Bound proteins were eluted with imidazole, buffer exchanged using Hi-Trap desalting columns (GE Healthcare), and collected in elution buffer (20 mM Tris-HCl, pH 7.6, 100 mM NaCl, 1 mM DTT). Aliquots were immediately frozen in liquid N_2_ and stored at −80°C.

### In vitro ubiquitination assay

Recombinant rabbit E1, UBCH5a and Ubc4 were kind gifts from Dr. Ben Distel (University of Amsterdam Medical Center, The Netherlands). Briefly, reactions were carried out at 37°C for 2 h in 20-μl reactions containing 25 mM Tris, pH 8, 100 mM NaCl, 5 mM MgCl_2_, 1 mM DTT, and the following as indicated: 5 mM ATP, 0.4 μg of recombinant rabbit E1, 0.4 μg of UBCH5a or Ubc4, 2.5 μg of ubiquitin (Biomol), and 0.4 μg of RNF145 RING. Reactions were stopped by addition of SDS-PAGE loading buffer and subjected to immunoblotting as described below.

### Adenovirus particle production

A sequence predicted to target both mouse and rat *Rnf145* and confer effective silencing (5’-GACGAAGCAGATCTGGCTC-3’) was cloned into pENTR/pTER+ (Addgene, 430–1) and subsequently transferred into pAd/BLOCK-iT™-DEST vector using gateway recombination (Invitrogen). Adenoviral particles were produced by transfecting PacI linearized plasmid into HEK293AD cells as previously reported [14], and subsequently amplified and titered (Viraquest, USA).

### Cell culture and transfections

RAW264.7, HEK293T, HeLa, and HepG2, THP1 cells were obtained from the American Type Culture Collection (ATCC). Cells were maintained in DMEM supplemented with 10% FBS at 37°C and 5% CO_2_. IHH cells were a kind gift from Dr. Geesje Dallinga-Thie (AMC, The Netherlands) and cultured in William’s E medium supplemented with 2 mM Glutamine, 10% FBS, 20 mU/ml bovine insulin and 50 nM Dexamethasone as previously described [29]. A431 cells were a kind gift from Elina Ikonen (University of Helsinki). Wild-type, and Lxrαβ^(-/-)^ MEFs were previously described [14]. HeLa cells with a stable integration of inducible control or *RNF145* shRNAs were generated by transducing cells with pLKO-3xLacO (Sigma) derived viral particles with control or *RNF145* targeting shRNAs. Cells were subsequently selected with puromycin (Sigma). To induce silencing of *RNF145* cells were cultured with 1 mM IPTG for 48 hours. The *RNF145* shRNA target sequence was 5’-AGGTGATTATTGAGTCTTGTA-3’. Where indicated, cells were depleted of sterols by culture in sterol-depletion medium (DMEM supplemented by 10% LPDS, 5 μg/ml simvastatin, and 100 μM mevalonate). To induce LXR signaling cells were cultured with 1 μM GW3965 (GW), 2.5 μM 22(*R*)-hydroxycholesterol (22OH), or 5 μM desmosterol. HEK293T, HeLa, A431, and HepG2 cells were transfected with the indicated amounts of plasmids using the JetPrime reagent (Polyplus). Transfection efficiency was monitored by co-transfecting an expression plasmid for GFP and was consistently >80% in HEK293T and HeLa cells. For silencing expression of *RNF145* we transfected HepG2 cells with 30nM of ON-TARGETplus SMART pool control (D-001810-10) or RNF145 (L-007146-00) using Lipofectamine RNAiMAX (Invitrogen). For live cell imaging HeLa cells were transfected with expression plasmids for mRnf145-eGFP and Calnexin-mCherry at a 5:1 ratio. Live cells were imaged 48 hours post transfection with a Leica TCS SP8 SMD.

### Animal experiments

Liver samples from *Lxrα*^*(-/-)*^ animals that were fed a standard laboratory chow diet (RMH-B; ABdiets, Woerden, The Netherlands) with or without T0901317 (Sigma-Aldrich, St. Louis, MO, USA; 0.015% wt/wt; 30 mg/kg) were obtained from Dr. Albert K. Groen (Academic Medical Center, Amsterdam] [31]. Rat primary hepatocytes were isolated and cultured as described [32]. Bone marrow cells were isolated from femurs and tibiae of four wild-type mice and four *Lxrαβ*^*(-/-)*^ mice following standard procedures [14]. In brief, mice cells were isolated and cultured in RPMI (Gibco) with 10000 U/ml penicillin/streptomycin (Gibco), 10% FBS (Gibco) and 15% L929-conditioned medium for 8 days to obtain bone marrow derived macrophages (BMDM) that were seeded 24 hours before the indicated treatments at a density of 1.5 × 10^5^ cells/cm^2^. Mouse tissues for total RNA isolation were collected from 3–6 male C57Bl/6 mice at 12 weeks of age. Handling and euthanasia of mice by means of CO_2_ asphyxiation were according to institutional guidelines and regulations, and all efforts were made to minimize suffering. Approval for these experiments was obtained prior to conducting the experiments from the institutional ethical committee on animal experimentation (IVD; Instantie Voor Dierenwelzijn of the Academic Medical Center of the University of Amsterdam).

### Dual-luciferase reporter assays

HEK293T cells were transfected with a firefly luciferase reporter plasmid (pGL2 Sv40 min or pGL3 basic, as indicated), a Renilla reporter plasmid pTK-RLUC (Promega), and as indicated with pCMX-LXR*α* and pCMV-RXR*α*, or empty pCMX. 48h after transfection cells were treated with vehicle (DMSO) or 1 μM GW3965 for 24 hours. Subsequently, the cells were washed twice in PBS and lysed in passive lysis buffer following the manufacturers instructions. The samples were then measured using the Dual-Luciferase Reporter Assay System (Promega) on a Glowmax Multi detection system (Promega) according to the manufacturer’s protocol. Each experiment was repeated at least three times in triplicate.

### Antibodies and immunoblot analysis

Cell lysates were prepared in RIPA buffer (150 mM NaCl, 1% Nonidet P-40, 0.1% sodium deoxycholate, 0.1% SDS, 100 mM Tris-HCl, pH 7.4) supplemented with protease inhibitors. For HMGCR and RNF145, sodium deoxycholate was added to a final concentration of 10% to the lysis buffer to prevent aggregation. Lysates were cleared by centrifugation at 4°C for 10 min at 10,000 g. Protein concentration was determined using the BCA assay with BSA as reference. Samples (10–40 μg) were separated on NuPAGE BisTris gels and transferred to nitrocellulose. Membranes were probed with the following antibodies: LDLR (Abcam, clone EP1553Y, 1:4000), tubulin (Sigma, clone DM1A, ascites fluid, 1:5000), ABCA1 (Novus Biologicals, NB400-105, 1:1000), FLAG (Sigma, clone M2, 1:1000), GFP (Santa Cruz sc-9996, 1:500), Myc (Santa Cruz 9E10, 1:3000), HA (Covance, clone 16B12, ascites fluid, 1:6000), HMGCR (rabbit polyserum was a kind gift from Dr. Peter Edwards, UCLA), SREBP2 (BD Biosciences, clone 1C6, 1:1000), SREBP1 (ThermoFisher, clone 2A4, 1:1000), actin (Merck Millipore, clone C4, 1:5000), V5 (Invitrogen, 46–0705, 1:3000), RNF145 (Abgent AP18281b, 1:8000), and ubiquitin (Enzo life Sciences, clone FK2, 1:1000). Secondary HRP-conjugated antibodies (Zymed Laboratories Inc.) were used and visualized with chemiluminescence on a Fuji LAS4000 (GE Healthcare). Unless indicated, blots shown are representative of at least 3 independent experiments with similar results.

### ChIP-seq analysis

Data analysis was performed using bowtie and HOMER [33] on previously published ChIP experiments: GSE28319 describing genomic binding sites of LXRα in human macrophages and GSE50944 for RAW macrophage-like murine cells [34,35]. The sequencing experiments were normalized to a total of 10^7^ uniquely mapped tags and visualized by preparing custom tracks for the UCSC Genome Browser.

### RNA isolation and quantitative PCR

Total RNA was isolated from cells using the Direct-zol™ RNA MiniPrep kit (Zymo Research and 1μg was reverse-transcribed with random hexamers using iScript (Bio-Rad). Real-time quantitative PCR assays were performed on a Lightcycler 480 II apparatus (Roche Applied Science) using Sensifast SYBR green (Bioline). Values were normalized to *36B4* and are shown as mean ± SD. Primer sequences are available upon request.

### Statistical analysis

**Fig 1:**
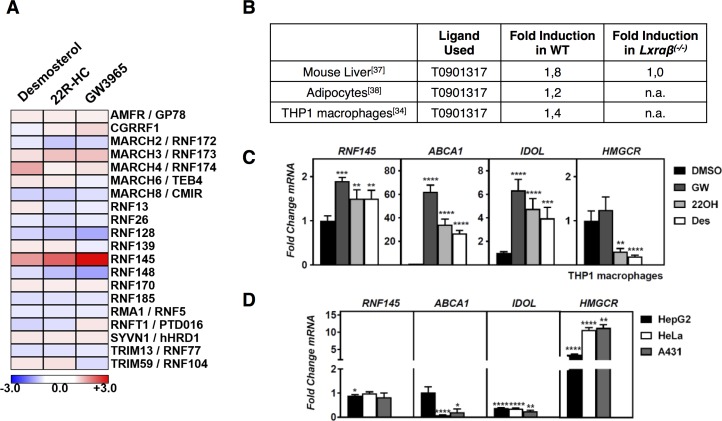
Identification of RNF145 as an LXR target. (***A***) Heat map presentation of the expression of predicted ER-resident E3 ubiquitin ligases in THP1 in response to treatment with desmosterol, 22*R*-HC, and GW3965 **[39]** (***B***) CHIP-seq experiments in which an LXR-ligand-responsive peak in the proximal promoter region of *RNF145* was detected are shown. The fold-induction by the LXR synthetic ligand, T0901317, that was subsequently determined by transcriptomic analysis is indicated. The response in livers of *Lxrα*^*(-/-)*^ was also evaluated; n.a, not available. (***C***) THP1 cells were grown in sterol-depletion medium and exposed to different LXR ligands for 6 hours: 1μM GW3965 (GW), 5μM 22(R)-hydroxycholesterol (22OH), 5μM Desmosterol (Des), or to vehicle (DMSO). Expression of the indicated genes was determined by qPCR and expressed as fold changes relative to vehicle control. Bars show mean ± SD and significant differences with vehicle are indicated (n = 4). (***D***) The indicated cells lines were incubated for 16 hours with sterol-depletion medium. Expression of the indicated genes was determined by qPCR and graph expresses fold change of sterol-depleted cells over complete medium. Bars show mean ± SD and significant differences from a value of 1 corresponding to no change (n≥4).

**Fig 2:**
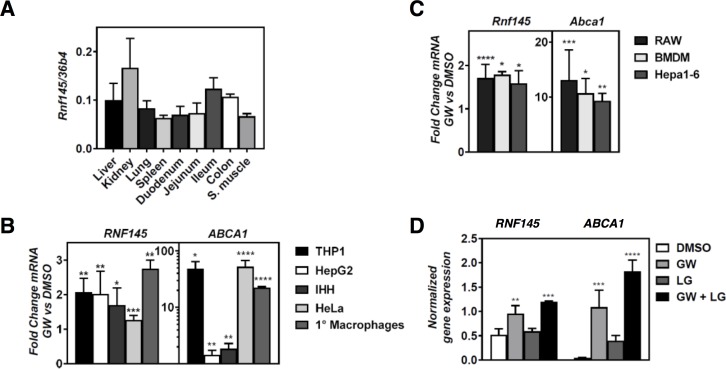
RNF145 is expressed in mouse tissues and is broadly regulated by LXR activation. (***A***) Expression of *Rnf145* was evaluated in the indicated mouse tissues by qPCR. Bars indicate mean ± SD (n = 3) (***B*,*C***) The indicated (***B***) human and (***C***) mouse cell lines and primary macrophages were cultured in lipoprotein-depletion medium for 16 hours and subsequently treated for 6 hours with 1μM GW3965 (GW). (***D***) THP1 cells were cultured in sterol depletion medium for 16 hrs and then treated with 1μM GW3965 (GW) and 100nM LG100268 (LG) for 6 hrs as indicated. Subsequently, *ABCA1* and *RNF145* expression was determined by qPCR and each bar and error represents the mean fold-change of ligand-treated cells over sterol-depleted cells ± SD (n≥3).

A one-way ANOVA test was performed with Holm-Sidak’s multiple comparison tests and single-pooled variance (Figs 1B and 4B). A one-sample t-test was performed to assess the difference with a theoretical mean of 1 (Figs 1C, 2A and 2B). An unpaired Mann-Whitney test with Welch’s correction was preformed to establish the significance of the difference between shScramble and shRnf145 for each gene (S1 Fig). In the other Figures two-way ANOVAs with Sidak’s multiple comparisons tests was used to establish statistical significance. Error bars indicate standard deviation (SD). Statistical significant *p* values are indicated by * *p*< 0.05, **p < 0.01, ***p < 0.001 and ****p<0.0001.

## Results

To identify novel ubiquitin-proteasomal-system components that may contribute to cellular cholesterol handling we reasoned that their transcript would be sterol-responsive, and as such be sensitive to manipulation of the LXR and SREBP pathways. To evaluate this we analyzed reported transcriptomic studies, ours included, in which the response to synthetic LXR ligands were examined [34,36–38]. Additionally, we recently reported a RNAseq study evaluating the transcriptional response of THP1 macrophages to distinct classes of LXR ligands: Synthetic (GW3965; GW), oxysterol (22(*R*)-hydroycholesterol; 22R-HC), cholesterol biosynthesis intermediate (desmosterol; Des) [39]. Given the central role of the ER as a hub for regulation of cellular cholesterol homeostasis, we primarily focused on E3 ligases, which are predicted to reside in this organelle [22]. Of the 24 predicted ER ligases, 20 were expressed in THP1 cells, only expression of *RNF145* responded to LXR activation by the three distinct classes of LXR ligands (Fig 1A). Next to established LXR-regulated genes such as *ABCA1*, *ABCG1*, and *IDOL*, this also resulted in identification of *RNF145*, a predicted E3-ubiquitin ligase, as an LXR responsive gene (Fig 1B and 1C). Typically, sterol depletion of cells results in attenuated LXR signaling. This has been attributed to decreased production of cholesterol biosynthesis intermediates, which at least in macrophages were proposed to be dominant LXR ligands [9,40]. Accordingly, expression of *IDOL* and *ABCA1* is decreased upon sterol depletion (Fig 1D). In contrast to this, expression of *RNF145* remained largely unchanged by sterol depletion, despite being responsive to LXR activation. This suggests that transcriptional regulation of *RNF145* may also integrate additional signals independent of LXRs. Expression of *Rnf145* in mouse tissues is ubiquitous and with comparable expression level, including in macrophage-rich tissues like the spleen (Fig 2A). Therefore, to evaluate whether *RNF145* was regulated by LXR in cells other than THP1 cells, we tested the response to LXR ligands in a panel of primary cells and cell lines of both human and murine origin. Similar to the finding in THP1 cells, activation of LXRs in this broad panel of cell types resulted in induction of *RNF145* expression with a magnitude of ~ 2.5-fold (Fig 2B and 2C). Since LXR forms a permissive heterodimer with RXR we also evaluated whether expression of *RNF145* is RXR-responsive (Fig 2D). As anticipated, expression of *ABCA1* was increased by the RXR ligand LG100268 (LG) alone, an increase that was further enhanced when cells were also treated with the LXR ligand GW3965. Yet unlike *ABCA1*, expression of *RNF145* did not significantly respond to RXR activation, possibly reflecting the narrow magnitude of *RNF145* mRNA regulation by LXR.

**Fig 3:**
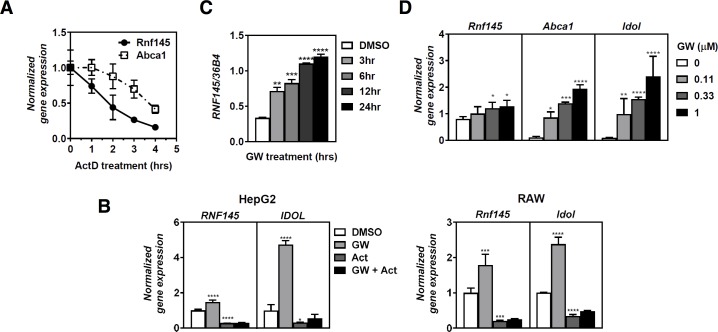
Characterization of ligand-induced expression of *RNF145*. (***A***) RAW264.7 macrophages were treated with 5μg/mL Actinomycin D (ActD) for the indicated time and expression of *Abca1* and *Rnf145* was determined by qPCR and plotted as mean ± SD relative to untreated cells (n = 3), (***B***) HepG2 and RAW264.7 cells were treated with 1μM GW3965 for 6 hours in the presence or absence of 5μg/ml actinomycinD for 4 hours, after which expression of the indicated genes was measured by qPCR. Bars indicate mean ± SD (n = 3) (***C*,*D***) THP1 macrophages were cultured in sterol-depletion medium for 16 hrs and then treated with (***C***) 1μM GW3965 (GW) for the indicated time, or (***D***) with the indicated concentration of GW3965 for 4 hrs. Subsequently, gene expression was evaluated qPCR and bars indicate mean ± SD (n = 3)

**Fig 4:**
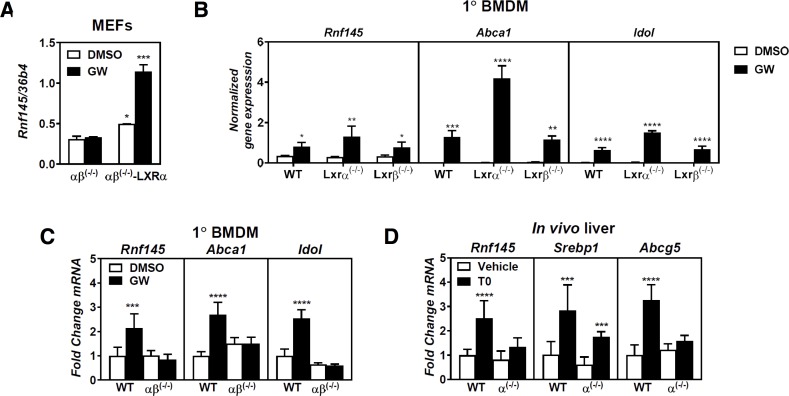
LXR-dependent regulation of RNF145. (***A***) Mouse embryonic fibroblasts from *Lxrαβ*^*(-/-)*^ mice or the same cells with stable overexpression of *LXRα*, or (***B***) bone-marrow-derived macrophage from wildtype, *Lxrα*^*(-/-)*^, and *Lxrβ*^*(-/-)*^ mice were cultured (***A*,*B***) in sterol-depletion medium for 16 hrs and subsequently treated with 1μM GW3965 (GW) for 6 hours. Expression of indicated genes was analyzed by qPCR and each bar shows the mean ± SD. Statistical significant differences from (***A***) *Lxrαβ*^*(-/-)*^ cells or (***B***) control treated cells are shown (n = 3). (***C***) Bone marrow-derived macrophages were isolated from either wildtype (WT) or *Lxrαβ*^*(-/-)*^ mice, cultured in sterol-depletion medium overnight and treated with 1μM GW3965 (GW) for 6 hours. Bars show the expression of the indicated genes relative to vehicle control ± SD (n = 4). (***D***) Expression of the indicated genes was analysed by qPCR in liver samples from WT and *Lxrα*^*(-/-)*^ mice that had either been treated with vehicle control or with 0,015% T0901317 in the diet for 14 days. Bars show mean gene expression relative to vehicle control ± SD (n = 6).

We then proceeded to investigate the mode of *RNF145* regulation by LXRs. A recent study suggested that *RNF145* transcript is short lived and is further decreased (~50%) in Jurkat cells treated with phorbol esters, largely as a result of the release of 3’-UTR-asssociated RNA-binding proteins [41]. In line with this report we found that the half-life of *RNF145* mRNA was indeed shorter than that of *ABCA1* (2.1 ± 0.3 vs. 3.6 ± 0.2 hours, *p<0*.*01*; Fig 3A). However, in both HepG2 (hepatic-like) and RAW (macrophage-like) cells the increase in expression of *IDOL*, an established LXR target gene, and of *RNF145* by pharmacological activation of LXR with the synthetic ligand GW3965 was fully blunted by actinomycin D (Fig 3B). Furthermore, the transcriptional response of *RNF145* to LXR activation was both time- and dose-dependent (Fig 3C and 3D). Together with results in Fig 2, this suggests that as a primary mechanism, activated LXRs do not modulate expression of *RNF145* by stabilizing its transcript, but rather by increasing its transcription. To demonstrate that regulation of *RNF145* by GW3965 is LXR-dependent we made use of fibroblasts derived from *Lxrαβ*^*(-/-)*^ mice (*αβ*^*(-/-)*^) or the same cells after they had been engineered to stably express *LXR****α*** (*αβ*^*(-/-)*^- *LXR****α***). In line with LXR-dependent regulation of *Rnf145* introducing back *LXR****α*** into these cells increased the basal expression of *Rnf145*, which was further increased by the synthetic ligand (Fig 4A). To investigate whether *Rnf145* is regulated in an isoform-specific fashion by LXRs we evaluated its induction in primary bone-marrow-derived macrophages from wildtype and isoform-specific knockout mice (Fig 4B). In both *Lxr****α***^*(-/-)*^ and *Lxr****β***^*(-/-)*^ macrophages *Rnf145* expression was induced by LXR activation indicating that both isoforms can regulate *Rnf145* expression. We extended these studies to primary bone-marrow-derived macrophages from wildtype and *Lxr****αβ***^*(-/-)*^ mice. As in the fibroblasts, induction of *Rnf145* was lost in macrophages from LXR-null mice further demonstrating that its regulation is LXR-dependent (Fig 4C). Finally, we also addressed regulation of *Rnf145* by an LXR ligand *in vivo*. As *Lxr****α*** is the main form found in the liver and *Rnf145* is detected in this organ (Fig 2A), we compared hepatic expression of *Rnf145* in wildtype and *Lxr****α***^*(-/-)*^ mice following a 14-day treatment with the synthetic LXR ligand T0901317 [31]. Similar to *Srebp1c* and *Abcg5*, two established hepatic targets of LXR, expression of *Rnf145* was increased by T0901317 in livers of wild-type mice, but not in those of *Lxr****α***^*(-/-)*^ mice (Fig 4D). Collectively, our results demonstrate that *RNF145* is a transcriptional target of LXR in human and rodent cells and also *in vivo* in mice, and that this regulation is LXR-dependent.

**Fig 5:**
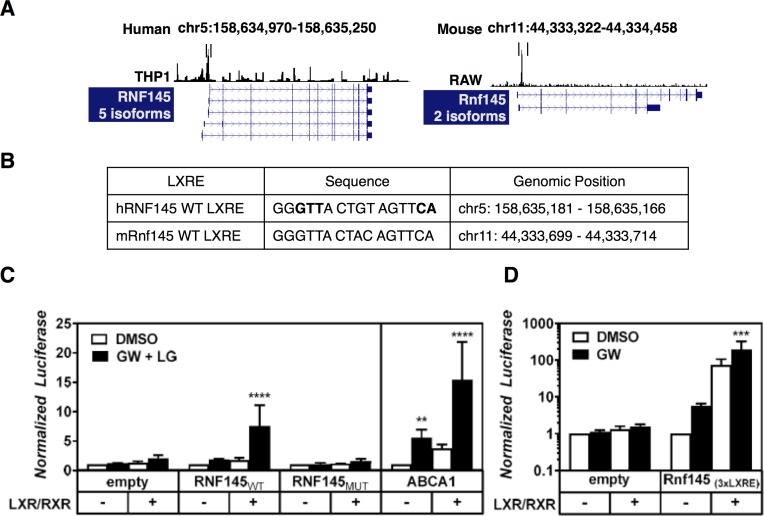
LXRE-dependent regulation of RNF145 expression by LXR. (***A***) LXR ChIP-seq experiments in human THP1 cells (GSE28319) and RAW macrophage-like cells (GSE50944) were analyzed and used to identify active LXREs within the *Rnf145/Rnf145* loci, as graphically illustrated. (***B*,*C***) Genomic location of the identified LXREs. In bold, nucleotides that were mutated to disrupt LXR binding (***C***) A 1kb genomic region upstream of the transcriptional start site of *hRNF145* was cloned into a pGL3basic. The putative LXRE was also mutated as indicated above. The empty, RNF145_WT_, RNF145_MUT_, and ABCA1 reporter plasmids were co-transfected with or without RXRα and LXRα expression plasmids in HEK 293T cells. 24 hours post-transfection the cells were treated with 1μM GW3965 (LXR) and 100nM LG100268 (RXR) for 24 hours and measured for luciferase signal (n≥3). (***D***) Cells were transfected with an empty or a tandem LXRE-containing pGL2 as in ***C***. In all luciferase experiments the transfection efficiency was normalized to co-transfected Renilla luciferase. Bars report normalized chemiluminescence relative to untreated control ± SD (n = 3).

LXRs activate transcription of their target genes by binding to LXREs, often present within, or in the vicinity of the regulated loci [4]. To identify a potential LXRE in the *RNF145* locus we analyzed published LXR ChIP-seq experiments done in RAW macrophage-like cells and in THP1 macrophages and mapped them to the UCSC genome browser [34,35]. In experiments from both mouse and human cells we observed a strong LXR-associated peak ±330 base pairs upstream of the transcriptional start site of *hRNF145*, which is homologous to a region within intron 1 of *mRnf145* (Fig 5A). We cloned the proximal promotor of *hRNF145* (1000 base pairs upstream of the transcriptional start site) and evaluated its ability to drive expression of a luciferase reporter (Fig 5B and 5C). Consistent with the cloned region containing an LXRE, promotor activity was increased by a combination of LXR/RXR ligands when LXR and the obligatory heterodimer partner RXR were introduced into cells, similar to what was observed with an *ABCA1*-promotor luciferase construct (Fig 5C). Importantly, mutating the predicted LXRE in the *hRNF145* promotor construct abolished regulation of the reporter element by LXR indicating that we have uncovered a functional LXRE. We also evaluated the corresponding predicted LXRE in the mouse genome by cloning it in tandem and testing whether it is able to enhance expression of luciferase driven by a minimal SV40 promotor (Fig 5D). Similar to the human genomic LXRE-containing region, the tandem LXRE construct readily induced expression of the reporter construct in response to both LXR/RXR and LXR ligand. These studies therefore identify a conserved LXRE in the human and mouse *RNF145/Rnf145* locus that provide a transcriptional basis LXR-dependent regulation.

**Fig 6:**
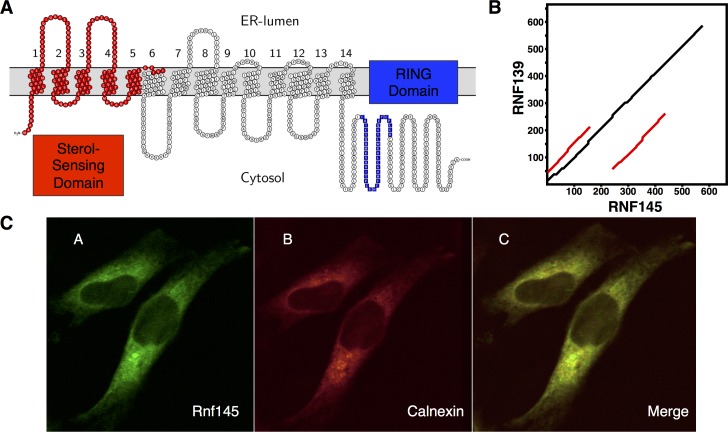
RNF145 is localized to the ER. (***A***) The secondary structure of RNF145 (NP_001186312) as predicted by CCTOP is shown. The first five N-terminal transmembrane helices are predicted to contain a sterol-sensing domain **[45]**. The C-terminal sequence contains a predicted RING structure. (***B***) An alignment dot plot between RNF139 and RNF145 was generated using PLALIGN **[55]**. The black line shows similarities between protein sequences. The red lines show repeating sequence motifs within the proteins. (***C***) HeLa cells were transfected with mRnf145-eGFP (A’,left) and Calnexin mCherry (B’,middle). Merged image is shown (C’, right). Images are representative of three independent experiments.

*RNF145* encodes an E3 ubiquitin ligase, and is predicted by Constrained Consensus TOPology prediction server (CCTOP) to contain 14 transmembrane spanning helices (Fig 6A), with both C- and N-termini facing the cytoplasm [42]. *In silico* analysis of the RNF145 sequence revealed that, next to a C-terminal RING domain, the first 6 transmembrane helices harbor a putative sterol-sensing domain (SSD), similar to that found in other sterol-sensing proteins as SCAP, NPC1 and PATCHED [43–45]. Within this SSD a sequence motif (Y-I/L-Y-F) implicated in Insig-binding in SCAP and HMGCR is also predicted [46]. These sequence elements are in line with RNF145 having a role in sterol metabolism and with regulation of the gene by LXRs. Further supporting this notion is the fact that sequence homology indicates RNF139/TRC8 as the closest RNF145 related protein. RNF139 and RNF145 are highly similar, differing largely in a C-terminal extension in RNF145 not present in RNF139 (Fig 6B). RNF139 is an ER-resident E3 ligase and has been implicated in controlling processing of SREBP2 and in sterol-stimulated degradation of HMGCR [24–26]. The localization of RNF145 is not known and a recent survey suggests RNF145 localizes to the ER [22]. We tested this directly by transfecting HeLa cells with an mRnf145-GFP expression construct along with Calnexin-mCherry, the latter to mark the ER compartment. We observed close co-localizaton of the two signals indicating that similar to RNF139, RNF145 is an ER resident E3 ligase (Fig 6C).

The SSD of RNF139/TRC8 is required for it ability to regulate SREBP processing [25,26]. As such, the high sequence homology with RNF145 and the presence of a predicted SSD in the N-terminal region of RNF145 prompted us to evaluate whether similarly, RNF145 can regulate of SREBP processing or signaling. To test this possibility we monitored the proteolytic processing of SREBP2 to its mature form in HeLa and HepG2 cells. As expected, sterol-depletion enhanced maturation of SREBP2 and increased the level of the LDLR and HMGCR, reflecting enhanced SREBP2 signaling (S1A Fig). Despite effective silencing of *RNF145* in these cells (S1B Fig), processing of SREBP2 to its mature form remained unaffected. Moreover, as assessed by qPCR analysis, silencing *RNF145* did not change the expression of SREBP2 target genes (S1A and S1B Fig). This was also the case in primary rat hepatocytes and mouse Hepa1-6 hepatocytes infected with an adenovirus encoding an shRNA targeting *Rnf145* (S1C Fig). Having ruled out an effect of RNF145 on SREBP2 signaling we evaluated its effects on SREBP1c, an established LXR target gene. SREBP1 processing requires both LXR and insulin signaling for maximal activation [47]. To evaluate SREBP1 processing we therefore starved HepG2 cells for 16 hours and evaluated whether silencing of *RNF145* influenced the response to insulin and pharmacological LXR activation (S1D Fig). The LXR ligand enhanced expression of *SREBP1c* and of Fatty Acid Synthase (*FASN)*, and this was further increased by insulin. However, these responses were insensitive to effective silencing of *RNF145* expression. Similarly, activation of *SREBP1c* and *FASN* remained intact in *RNF145*-silenced HeLa cells (S1E Fig) and in primary rat hepatocytes (S1F Fig). Collectively, our results suggest that the LXR-RNF145 axis does not seem to regulate SREBP1/2 processing under the experimental conditions we evaluated.

**Fig 7:**
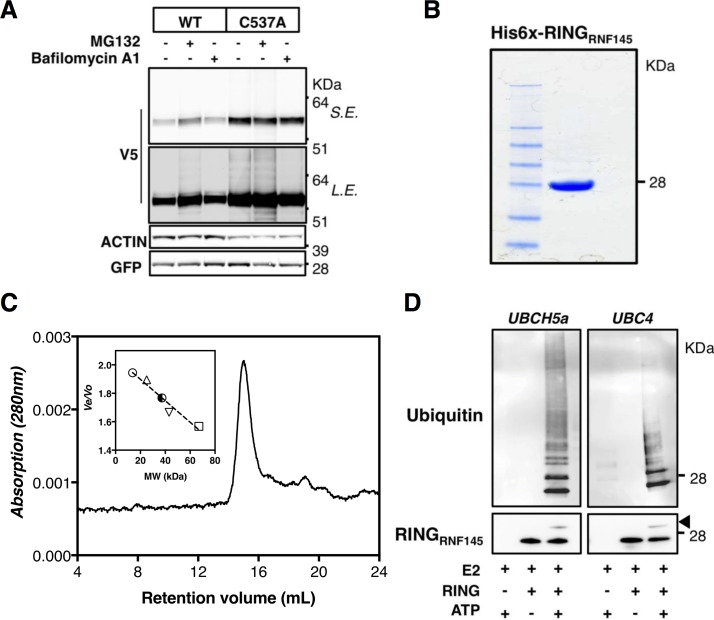
RNF145 has E3 ubiquitin ligase activity. (***A***) HEK293T cells were transfected with wildtype (WT) or RING-mutated (C537A) mRnf145-V5. Subsequently cells were treated with vehicle, 25μM MG-132, or 100nM Bafilomycin A1 for 4 hours. Total cell lysates were immunoblotted as indicated; S.E, short exposure, L.E, long exposure (n = 3) (***B***) Recombinant His6-RNF145 RING protein was purified and 1 μg loaded on SDS-PAGE gel. An image of a Coomassie Brilliant Blue-stained gel is shown. (***C***) Size Exclusion Chromatography elution profile of recombinant His6-RNF145 RING protein separated over a Superdex 75 10/300 column calibrated with a set of standard proteins. The elution volume corresponds to an apparent molecular mass of 39 kDa, indicating that in solution His6-RNF145 RING forms a dimer. (***D***) *In vitro* ubiquitination assays were done with the RNF145 RING protein in combination with the E2 enzymes UBCH5a and Ubc4. Reactions were carried at 37°C for 2 hours. Subsequently, reactions were immunoblotted as indicated. Arrow indicates an auto-ubiquitylation band of the RING of RNF145. Blots are representative of 2 independent experiments.

ERAD plays a critical role in controlling cholesterol homeostasis through regulated degradation of, amongst others, INSIGs and HMGCR [18]. The presence of a RING domain (Fig 6A), often found in E3 ligases, thus suggests that it may act as an ERAD-associated E3 ligase. To establish this we evaluated the E3 activity of RNF145. Many E3 ligases are short-lived proteins, owing to their intrinsic auto-ubiquitylation activity and subsequent degradation [48]. Accordingly, we find that blocking the proteasome with MG-132, but not the lysosome with Bafilomycin A1, led to stabilization of wild-type Rnf145 and appearance of a higher molecular-weight smear consistent with ubiquitylation (Fig 7A). Inline with auto-ubiquitylation and subsequent degradation of Rnf145, introducing a RING-disrupting mutation (C537A) markedly stabilized Rnf145 and eliminated further stabilization by proteasomal blockage (Fig 7A). To conclusively establish RNF145 as an E3 ligase we generated and purified a recombinant RNF145_RING_ peptide. The purified peptide migrated at a molecular weight slightly higher than its predicted weight (18 kD) on SDS-PAGE (Fig 7B) and in gel filtration eluted at a size consistent with it being a dimer in solution (Fig 7C). In conjunction with two E2 ubiquitin conjugating enzymes, UbcH5a and Ubc5, RNF145_RING_ stimulated robust ATP-dependent production of free poly-ubiquitin chains and of RNF145_RING_ auto-ubiquitylation (Fig 7D), establishing RNF145 as a *bona fide* E3 ubiquitin ligase. In aggregate, our results identify RNF145 as an ER-resident E3 ubiquitin ligase that is under transcriptional regulation by LXRs.

## Discussion

Cellular cholesterol levels must be tightly maintained and herein LXRs play a central role [4]. Therefore, determining the full set of genes that make up the LXR-regulated genetic program is an important facet of lipid metabolism. As such, the most important findings of this study are the identification of a novel LXR regulated target-gene, *RNF145*, and the demonstration that it localizes to the ER and possess E3 ubiquitin ligase activity.

The ubiquitin proteasomal system has been implicated in all cellular process and has been demonstrated to play a role, amongst others, in proteostasis, immunity, cancer, autophagy and transcription [17]. Specificity in this system is largely dictated by the ±700 E3 ubiquitin ligases predicted in the human genome. Their involvement in cholesterol metabolism, particularly owing to their ability to rapidly and acutely regulate cholesterol-related processes, is emerging [18]. To date only a handful of E3s have been directly associated with cholesterol metabolism. In these cases the E3s were demonstrated to regulate basal and stimulated degradation of key regulatory nodes of cholesterol metabolism including HMGCR (GP78, HRD1, TRC8, and MARCH6) [23,24,28,49], Squalene Epoxidase (MARCH6) [28,29], SREBP (TRC8, Fbw7 and RNF20) [21,25,26,50], and the LDLR (IDOL) [14]. The activity and expression of some of these ligases is also controlled by cholesterol (or metabolites thereof); cholesterol stimulates MARCH6-dependent ubiquitylation and degradation of Squalene Epoxidase [28], the stability of RNF139/TRC8 is also regulated by cholesterol [26], and *IDOL* is a sterol-responsive gene [14]. We demonstrate here that *RNF145* is a second LXR-regulated E3 ligase in a variety of cell types. We show that expression of *Rnf145* in mouse tissue is ubiquitous and of comparable level. However, a point warranting attention is that bioinformatic analysis of circadian expression of hepatic genes suggests a marked dark-light regulation of *Rnf145* with maximal expression preceding start of the dark cycle (CIRCADB; [51]). Furthermore, we identify an LXRE in both the murine and human *RNF145* locus and using *Lxr*-ablated cells show that regulation of *RNF145* expression by LXR ligands is LXR-dependent. However, unlike other LXR-regulated genes, expression of *RNF145* seems insensitive to the cellular sterol status and depletion of cellular cholesterol does not reduce *RNF145* expression. Divergent regulation of LXR target genes has been previously reported for *ABCA1* and *SREBP1C* and attributed to differential recruitment of transcriptional co-activators [52]. Alternatively, *RNF145* may be subject to LXR-independent transcriptional regulation that maintains its basal expression even in face of declining cellular sterol levels. A more intriguing possibility is that sterols not only induce expression of *RNF145*, but also regulate its activity or stability as shown for MARCH6 and TRC8, respectively [26,28]. This is in line with the presence of a predicted SSD in RNF145 that is homologous to the one found in TRC8. Unfortunately, the absence of antibodies able to detect endogenous RNF145 protein prevented us from evaluating the effect of sterols on protein stability. It is nevertheless tempting to speculate that sterols control the activity or abundance of RNF145 and that when elevated they also control its expression. This may offer a plausible explanation as to why the magnitude of RNF145 induction by LXR is limited in cells (2–3 fold) since this induction will be coupled with enhanced E3 activity, potentially also in conjunction with circadian regulation.

The regulation by LXRs, the presence of the SSD and the strong homology with TRC8 suggested to us that RNF145 could be involved in regulation of SREBP processing. However, our experiments in several cell lines and primary hepatocytes indicate that under the experimental conditions we evaluated this is not the case (S1 Fig). Two recent studies suggest RNF145 is involved in the regulation of the phagocytic oxidative burst in macrophages [53], or in activation of T cells by PMA [41] yet the molecular mechanisms behind these phenotypes is not fully elucidated. Our study adds to these observations and demonstrates for the first time that *RNF145* is subject to sterol-dependent regulation. *RNF145* is broadly expressed and regulated by LXR in a variety of cell types suggesting it may be part of the canonical LXR-dependent gene program.

In conclusion, next to IDOL we have identified a second sterol-regulated E3 ubiquitin ligase, RNF145, and discuss its potential role in lipid metabolism. The recent identification of an epigenome-wide association between methylation of *RNF145* and body-mass index in African American adults further supports a metabolic role for this E3 [54]. Additional studies are needed to establish proteins subject to RNF145-dependent ubiquitylation and to evaluate the role this plays in lipid metabolism.

## Supporting information

S1 Fig RNF145 does not influence SREBP processing. **(*A*,*B*)** HepG2 and HeLa cells were transfected with control (Ctrl) or *RNF145* (Kd) siRNAs. Subsequently, cells were cultured in sterol-depletion medium for 24 hours. Total cell lysates were immunoblotted as indicated and a representative blot of 3 independent experiments is shown, or expression of the indicated genes was evaluated by qPCR. Each bar and error represent the fold-change relative to control siRNAs ± SD. (***C***) Primary rat hepatocytes and Hepa1-6 cells were transduced at an MOI of 25 for 48h with adenoviruses encoding a control or *Rnf145* shRNA. Bars represent mean ± SD (n = 3). (***D***) HepG2 cells were transfected as indicated above and cultured in medium containing 5% BSA for 24 hours. Subsequently cells were treated with vehicle or 1 μM GW3965 (GW) for 6 hours with or without 100 nM insulin added in the last 30 minutes. Expression of the indicated genes was analyzed by qPCR and the bars show mean ± SD (n = 3). **(*E*)** HeLa cells with stable integration of an inducible control (scramble) or *RNF145* shRNAs were treated with 1 mM IPTG to induce silencing of *RNF145* for 48 hours. Subsequently, cells were cultured in lipoprotein-depletion medium for 16 hours and then treated with vehicle or 1μM GW3965 (GW) for an additional 6 hours. Expression of *RNF145* and *FASN* is shown and the bars represent fold changes relative to non-treated control cells ± SD (n = 3). (***F*)** Primary rat hepatocytes were transduced with adenoviruses as indicated in ***C*** and expression of the indicated genes was evaluated by qPCR. Bars represent mean ± SD relative to control cells (n = 3).
(PDF)

## Abbreviations

SREBP: Sterol regulatory element binding proteins
LXR: Liver X Receptors
LDL: Low-density lipoprotein
IODL: Inducible degrader of the LDLR
ERAD: ER-associated degradation
HMGCR: 3-hydroxy-3-methyl-glutaryl-coenzyme A reductase
LPDS: Lipoprotein deficient serum
LXRE: LXR responsive element
22OH: 22(*R*)-hydroxycholesterol
SSD: sterol-sensing domain
