# Supplementary figures and images for: Identification of the ER-resident E3 ubiquitin ligase RNF145 as a novel LXR-regulated gene
Source: PLoS One. 2017 Feb 23;12(2):e0172721. doi: 10.1371/journal.pone.0172721 (PMC5322959; doi:10.1371/journal.pone.0172721)

S1 Fig

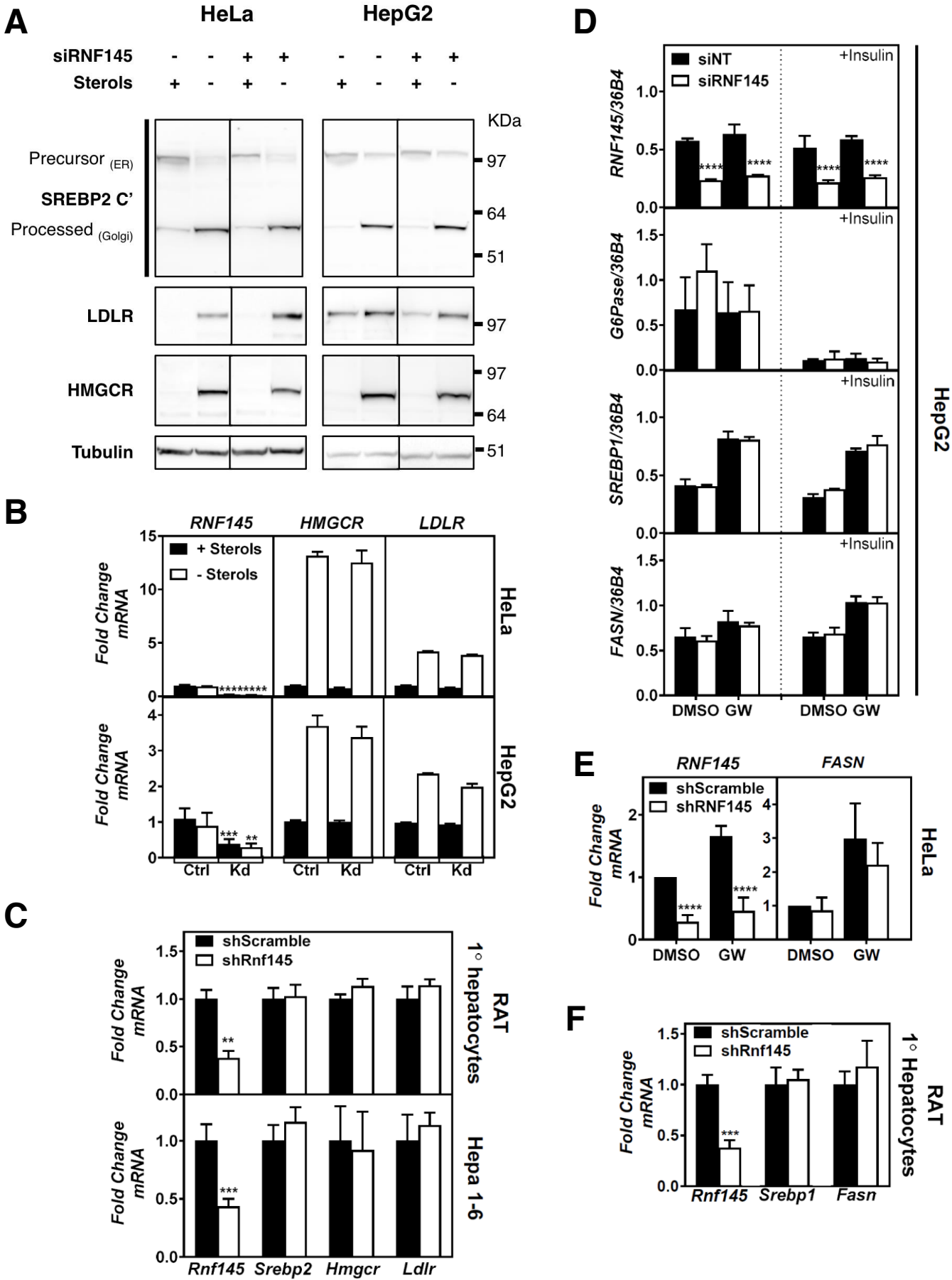

Supplement: S1 Fig — (A,B) HepG2 and HeLa cells were transfected with control (Ctrl) or RNF145 (Kd) siRNAs. Subsequently, cells were cultured in sterol-depletion medium for 24 hours. Total cell lysates were immunoblotted as indicated and a representative blot of 3 independent experiments is shown, or expression of the indicated genes was evaluated by qPCR. Each bar and error represent the fold-change relative to control siRNAs ± SD. (C) Primary rat hepatocytes and Hepa1-6 cells were transduced at an MOI of 25 for 48h with adenoviruses encoding a control or Rnf145 shRNA. Bars represent mean ± SD (n = 3). (D) HepG2 cells were transfected as indicated above and cultured in medium containing 5% BSA for 24 hours. Subsequently cells were treated with vehicle or 1 μM GW3965 (GW) for 6 hours with or without 100 nM insulin added in the last 30 minutes. Expression of the indicated genes was analyzed by qPCR and the bars show mean ± SD (n = 3). (E) HeLa cells with stable integration of an inducible control (scramble) or RNF145 shRNAs were treated with 1 mM IPTG to induce silencing of RNF145 for 48 hours. Subsequently, cells were cultured in lipoprotein-depletion medium for 16 hours and then treated with vehicle or 1μM GW3965 (GW) for an additional 6 hours. Expression of RNF145 and FASN is shown and the bars represent fold changes relative to non-treated control cells ± SD (n = 3). (F) Primary rat hepatocytes were transduced with adenoviruses as indicated in C and expression of the indicated genes was evaluated by qPCR. Bars represent mean ± SD relative to control cells (n = 3). (PDF) [file pone.0172721.s001.pdf]
